# True redo aortic root surgery after a Freestyle conduit failure: Use of an orthopedic shaver

**DOI:** 10.1016/j.xjtc.2026.102446

**Published:** 2026-05-28

**Authors:** Patrick D. Rudersdorf, William M. Whited

**Affiliations:** Division of Cardiothoracic Surgery, Department of Surgery, St Anthony Hospital, CommonSpirit, Lakewood, Colo

**Figure:**
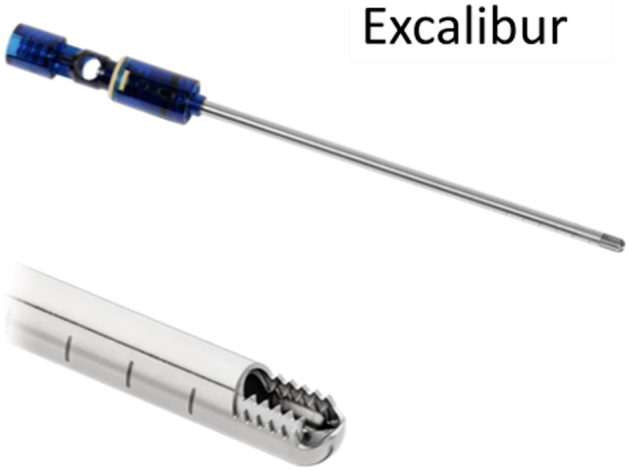
Excalibur 5.0-mm shaver.

**Central Message**

The management of intracardiac calcium is a technical challenge for cardiac surgeons. The Excalibur shaver (Arthrex) is a tool to add to the surgeon’s armamentarium for this challenging operation.

True-redo aortic root surgery for structural valve degeneration in the setting of a previous homograft or Freestyle xenograft is a technically demanding, high-risk operation, which is directly related to the degree of calcification. El-Hamamsy and colleagues^1^^,^^2^ have shown elevated calcium scores at 8 years, consistent with other studies in both graft types. Elimination of calcified tissue during redo surgery is mandatory; therein lies the challenge and the potential lethal complications, which have previously been well described in the literature.^3^ Some groups have shown acceptable mortality rates for redo surgery, whereas others have recommended transcatheter aortic valve replacement (TAVR) versus redo surgery, this despite 5-year survival rates being similar.^4^^,^^5^ Valve-in-valve TAVR carries the risk of annular rupture as well as coronary artery obstruction. The existence of a “calcium removal tool” would be an ideal instrument in this setting. Described techniques exist in the literature.^E1^

Here, we present the off-label use of a novel approach using an orthopedic shaver (Excalibur 5.0 mm; Arthrex) to excise and debride the calcified aortic root in a 57-year old patient with a previous 27-mm Freestyle porcine aortic root. The patient presented in acute, decompensated, congestive heart failure related to severe aortic valve regurgitation. Standard preoperative imaging, including a cardiac-gated computed tomography angiography scan, was only remarkable for a calcium score of 9585 (Figures 1 and 2).

**Figure 1:**
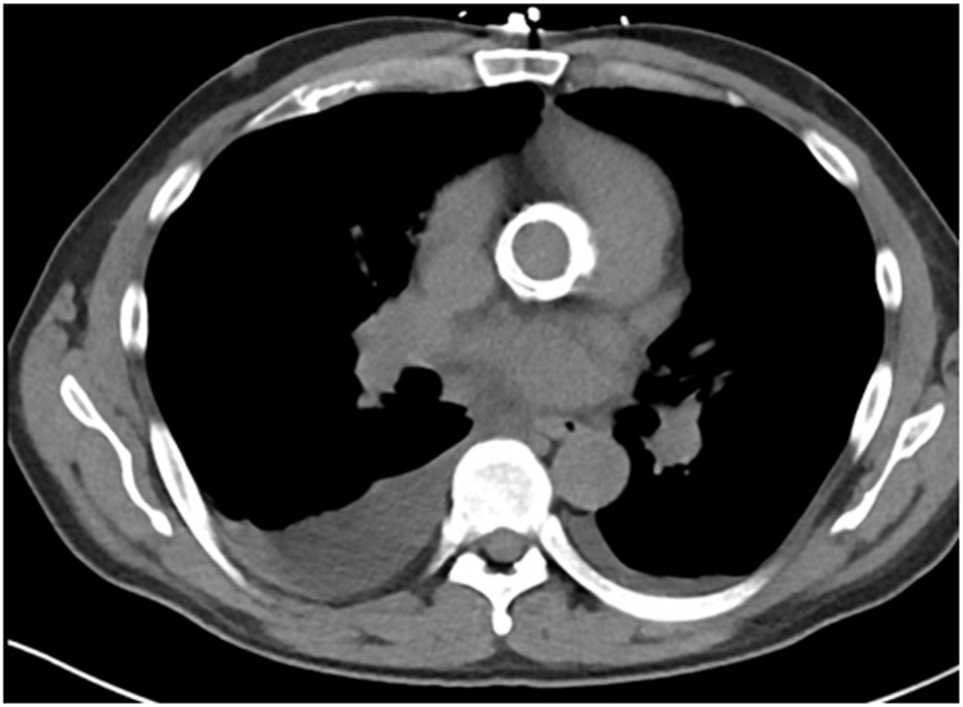
Preoperative CT scan. Axial image showing circumferential aortic root calcification. *CT*, Computed tomography.

**Figure 2:**
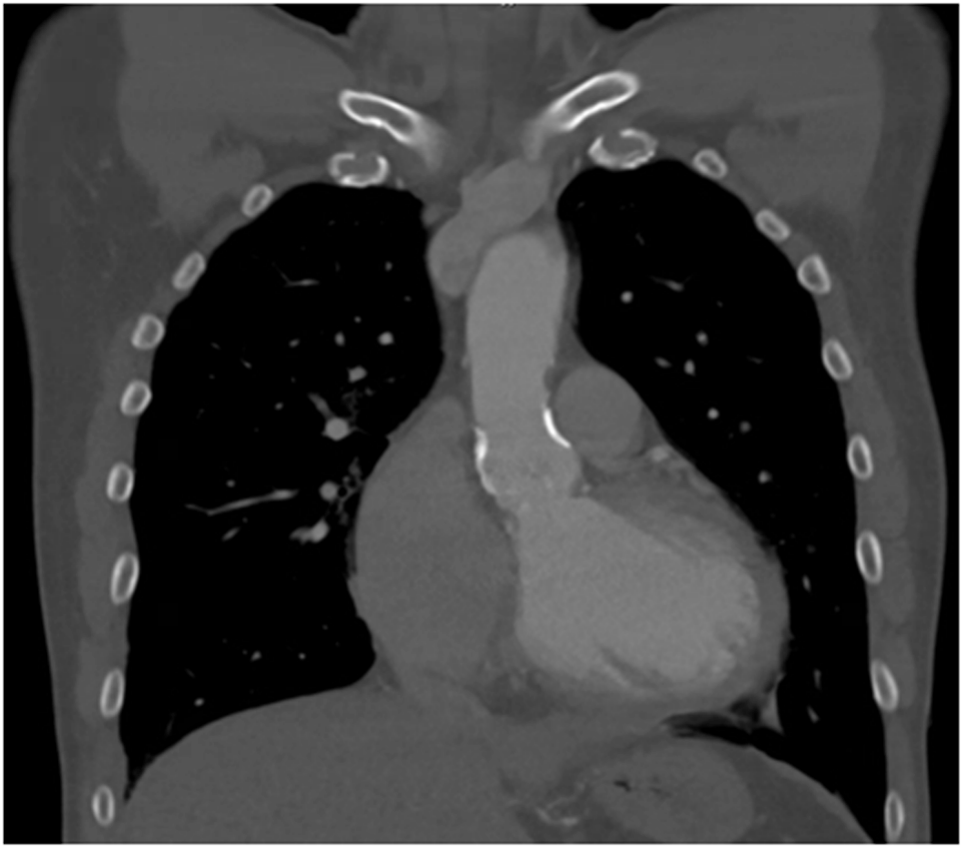
Preoperative CT scan. Coronal imaging showing calcification of the aortic root and proximal ascending aorta. *CT*, Computed tomography.

The patient provided consent to undergo a true redo aortic root replacement. Other well-described treatment options were considered; however, because of the patient's strong preference for a biologic surgical aortic valve replacement (SAVR), the operative surgeon believed a true redo aortic root would result in a better long-term prognosis by allowing for a 29-mm SAVR implantation and optimizing coronary heights. Because of the significant amount of calcification circumferentially at the level of the annulus, the intraoperative decision for an iso-SAVR was abandoned. Postoperatively, the patient experienced no complications and was discharged home postoperative day 8. Follow-up imaging showed a normally functioning 29-mm SAVR with a mean gradient of 6 mm Hg, and a computed tomography angiography scan showed a calcium score of 0 with no pseudoaneurysms (Figures 3 and 4). This technique and instrumentation will add to the surgeon's armamentarium and surgical literature for the management of intracardiac and vascular graft calcification.

**Figure 3:**
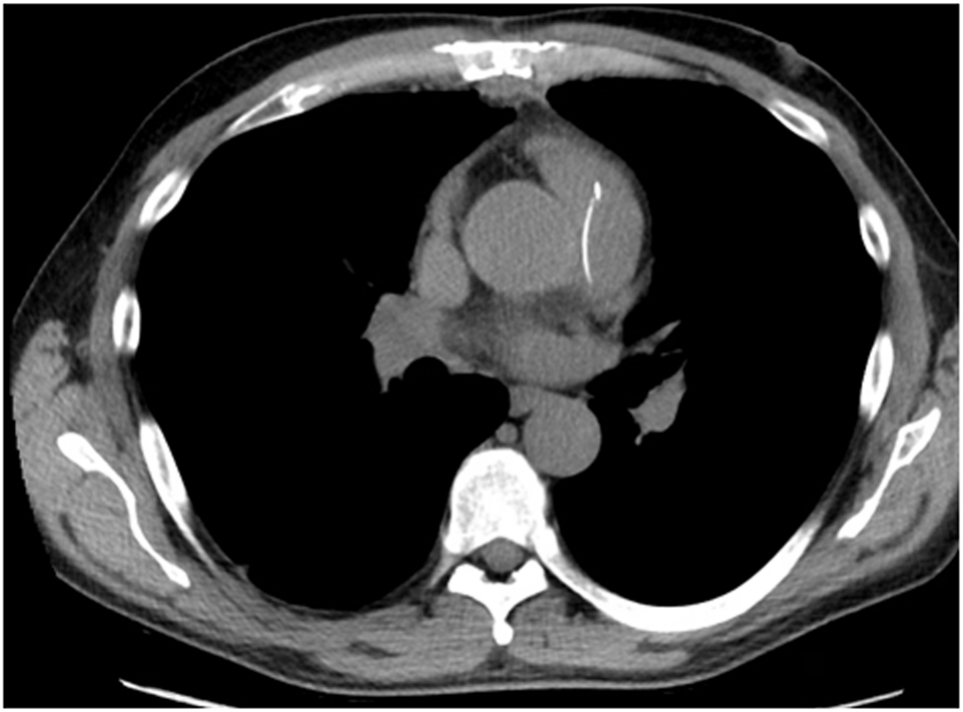
Postoperative CT scan, noncontrast. Axial image showing complete removal of calcium; calcium score is 0. *CT*, Computed tomography.

**Figure 4:**
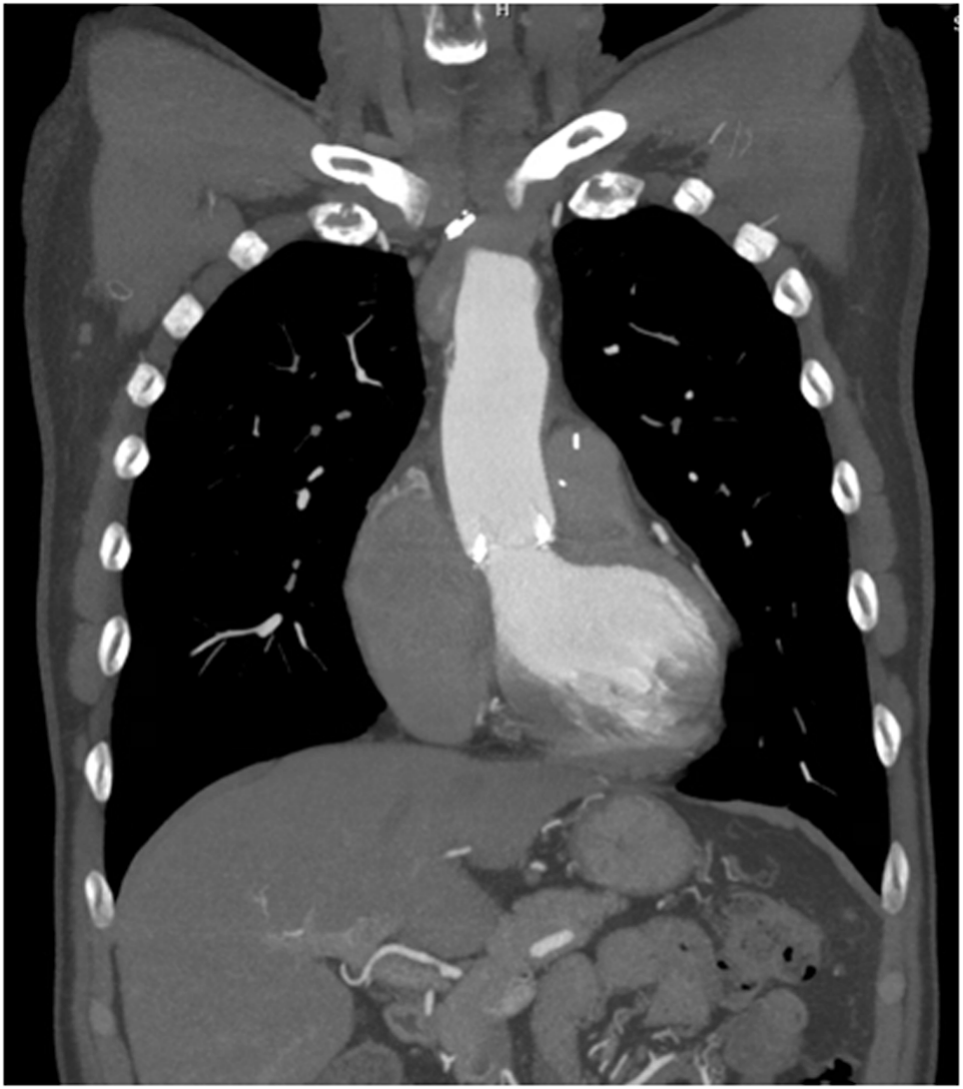
Postoperative CT scan. Coronal image showing SAVR and complete removal of calcium by Excalibur. *CT*, Computed tomography; *SAVR,* surgical aortic valve replacement.

This study was approved by the institutional review board (no. 2381236-1, approved October 30, 2025). This study met the criteria for a waiver of consent.

## Background

The management of intracardiac calcifications and calcified vascular grafts is a technical challenge for cardiac surgeons. The instrumentation currently at the surgeon's disposal has not changed for decades, which includes a scalpel, rongeurs, scissors, and Bovie electrocautery. CUSA (Clarity Ultrasonic Surgical Aspirator System; Integra LifeSciences) has been described as an adjunct to this; however, it too, has its own unique limitations.

Current techniques used by surgeons for all types of debridement of calcium include the combined use of rongeurs, forceps, and scalpel. Well described in the literature is the use of the ultrasonic oscillator for aortic stenosis. This has been an off-label use for decades and has been the only instrument that provides mechanical debridement for pathologic, calcified tissue in the heart. However, dangers exist with the use of this mechanical debridement for important reasons: (1) the tip on the CUSA is not protected; (2) calcified and healthy tissue alike are morcellated, having the unattended consequence of seriously damaging healthy tissue in proximity to the pathologic calcifications: and (3) it does not reliably eliminate the calcific debris from the surgical field, thus exposing the patient to risk of systemic embolization.

The ideal instrument for mechanical debridement of pathologic, calcified tissue in the heart should, among others, have these properties: (1) mechanical debridement of only calcified tissue; (2) produces no collateral damage to surrounding structures; and (3) reliably aspirates the calcific debris. The design of Excalibur is such that these are met because it’s designed for use in orthopedic surgery for precise shaving, that is, only calcified tissue is debrided. Furthermore, the mouth of the Excalibur is only 180° and is not present at the tip; thus, it is protective of surrounding structures. The Excalibur also has the effect of fracturing and loosening surrounding calcium, creating large, manageable portions that are easily removed. Finally, the wide mouth of the 5.0-mm Excalibur allows calcium debris to be concomitantly aspirated as is it debrided.

## Technique

Redo sternotomy was performed, with central cannulation followed by the commencement of cardiopulmonary bypass and cardioplegic arrest all in standard fashion. The aorta was transected. Upon inspection, there was obvious structural valve deterioration. The leaflets were resected to aid in visualization. On palpation, the entire Freestyle xenograft was calcified in a 360° fashion (Figures 1 and 3). In addition, the os of the left main and right coronary arteries were circumferentially calcified.

The entire aortic root was then completely mobilized circumferentially to the level of the native aortic valve annulus and the coronary arteries detached from the graft. Next, the Excalibur 5.0 mm × 13 cm (Arthrex), programmed to 8000 rpm and nonoscillatory settings, with maximal suction, was used to mechanically debride the calcified Freestyle xenograft. Excalibur's mouth was directed toward the area of calcium and by using foot pedal activation, the Excalibur was used to mechanically debride all calcified tissue. Only healthy, noncalcified tissue remained. It should be noted to provide irrigation with cold saline along the shaft of the Excalibur. From a technical standpoint, only gentle pressure on the Excalibur's handpiece is required during the debridement process.

Specifically, for this case the Excalibur was first used in the non-sinus of Valsalva and the calcium was debrided until healthy, native tissue was encountered. Once a plane of healthy tissue was identified, the Excalibur was then used to mechanically debride the calcified right and left sinuses, again, until healthy tissue was encountered. Rongeurs and scissors were used in conjunction during the debridement process, and the Excalibur advantageously produced large, manageable, calcium chunks; however, the Excalibur debrided the majority of the calcium and the xenograft root. Once debridement was completed, the native annulus was completely intact, all calcium was removed, and the aortic root and left ventricle were then copiously irrigated. Annular sutures were easily placed, and a Bio-Bentall (29-mm Inspiris with a 34-mm graft) was implanted in standard fashion, and the patient weaned from cardiopulmonary bypass. There was complete hemostasis, and on transesophageal echocardiography it was observed that the SAVR was functioning normally.

## Discussion

The management of calcified tissue represents a challenge for cardiac surgeons, especially a calcified aortic root requiring true redo aortic root surgery. Complete debridement of the calcium is a requisite for a successful operation. The Excalibur is a tool to add to the surgeon's armamentarium for this technically demanding operation because it very effectively results in complete debridement of pathologic calcium while simultaneously sparing healthy tissue.

Additional applications in cardiac surgery could include removal of calcium in native aortic valve stenosis and mitral annular calcification as well as TAVR explantation or other redo operations in adult patients with congenital heart disease. The author has extensive experience in all 4 aforementioned operations with more than 50 cases to date. Furthermore, its use in resection of myocardium in hypertrophic obstructive cardiomyopathy cases is another potential application. Further studies are needed.

## Conflict of Interest Statement

Dr Rudersdorf reported paid consultant for Gore and Arthrex. Dr Whited reported no conflicts of interest.

The *Journal* policy requires editors and reviewers to disclose conflicts of interest and to decline handling or reviewing manuscripts for which they may have a conflict of interest. The editors and reviewers of this article have no conflicts of interest.
